# Characteristic features of statistical models and machine learning methods derived from pest and disease monitoring datasets

**DOI:** 10.1098/rsos.230079

**Published:** 2023-06-28

**Authors:** Shigeki Kishi, Jianqiang Sun, Akira Kawaguchi, Sunao Ochi, Megumi Yoshida, Takehiko Yamanaka

**Affiliations:** ^1^ Research Center for Agricultural Information and Technology, National Agriculture and Food Research Organization 105-0003, 2-14-1 Kowa Nishi-Shimbashi Building, Nishi-Shimbashi, Minato, Tokyo, Japan; ^2^ Western Region Agricultural Research Center (Kinki, Chugoku and Shikoku Regions), National Agriculture and Food Research Organization 721-0975, 6-12-1 Nishi-Fukatsu, Fukuyama, Hiroshima, Japan; ^3^ Institute for Plant Protection, National Agriculture and Food Research Organization 305-8666, 2-1-18 Kannon-dai, Tsukuba, Ibaraki, Japan

**Keywords:** crop pest, crop disease, machine learning, statistical model

## Abstract

While many studies have used traditional statistical methods when analysing monitoring data to predict future population dynamics of crop pests and diseases, increasing studies have used machine learning methods. The characteristic features of these methods have not been fully elucidated and arranged. We compared the prediction performance between two statistical and seven machine learning methods using 203 monitoring datasets recorded over several decades on four major crops in Japan and meteorological and geographical information as the explanatory variables. The decision tree and random forest of machine learning were found to be most efficient, while regression models of statistical and machine learning methods were relatively inferior. The best two methods were better for biased and scarce data, while the statistical Bayesian model was better for larger dataset sizes. Therefore, researchers should consider data characteristics when selecting the most appropriate method.

## 1. Introduction

Crop diseases and pests can cause devastating outbreaks and substantially damage crop yields globally. Understanding their mechanisms of prevalence and predicting their occurrences are central for maximizing and stabilizing crop yields [1].

While statistical modelling approaches remain popular to predict the occurrences, increasing studies have used machine learning (ML) methods. In the conventional methods, statistical models are applied to a dataset that includes a sequence of historical occurrence records for a pest or disease [2,3]. The results are then compared, and the best-fit model is selected using an index of how well a model fits, such as the Akaike information criterion (AIC) [4]. The mechanism of pest dynamics is interpreted using selected parameters and the estimated values of the best-fit model. The best-fit model is expected to forecast future occurrences and abundances. Various statistical models have been developed and used depending on the data structure and purpose. A common feature of standard statistical methods is that a researcher determines the model structure before fitting the model to the data. For example, in a linear model, the relationship between explanatory and response variables is assumed to be linear, and the probability distribution of the response variable to be Gaussian. In a Bayesian model, the forecasting ability improved with the incorporation of variable and seasonality hierarchy [1,5]. However, this information may be subjective, incorrect and biased [6].

ML methods are also effective for forecasting the occurrences and abundances of crop diseases and pests [7–12]. Skawsang *et al*. [10] compared the forecasting efficiency of artificial neural network and random forest using the monitoring data of the brown plant hopper, *Nilaparvata lugens*, on rice in Thailand. They showed that the artificial neural network exhibited better prediction ability when the climate data and host plant phenology were considered. ML methods have been applied to pest management science and gradually to broader areas of agricultural science [13], such as forecasting crop yields [14,15], and detecting and recognizing pest and disease species [16,17].

ML methods are expected to provide more accurate predictions without prior information, while statistical models provide more interpretable results so that researchers can use them to suggest the inner mechanisms using prior information [18]. However, the performance of ML methods may depend on the data characteristics. When a dataset is small and the records are scarce, statistical models, such as the Bayesian model, may exhibit superior prediction skill than ML algorithms with the aid of the model structure. De Oliveira *et al*. [12] compared the results of multiple linear regression, k-nearest neighbour, random forest and neural network using the monitoring datasets of four pests and diseases on *Coffea arabica*. The random forest was the best when evaluated using R-square, the root mean square error (RMSE) and Willmott's *d,* while the multiple linear regression was the second or third best. The neural network was the worst in most cases. Though some studies have concluded that ML methods were superior to statistical models, especially with larger data sizes [8,10–12], they employed datasets of a limited variety of crops and pests. What data characteristics make ML methods advantageous in prediction remain unclear.

In this study, we explored characteristic features of two statistical and seven ML methods when making predicted values, using 203 monitoring datasets of crop pests and diseases (electronic supplementary material, table S1). The two statistical methods were Bayesian state-space model and multiple linear regression model, and the seven ML methods were lasso, elastic net, decision tree (DT), random forest (RF), k-nearest neighbour (kNN), support vector machine (SVM) and neural network (NN). The datasets contain the monitored abundance of pests and diseases on four major crops: cucumber, eggplant, tomato and strawberry. We assigned each dataset to all the statistical and ML methods and calculated the standardized value of RMSE. We then compared the value among the methods and discussed the effect of dataset characteristics on the prediction performance.

## 2. Methods

### 2.1. Dataset

The field monitoring survey was carried out by prefectural stations according to the national pest forecasting scheme of the Ministry of Agriculture, Forestry and Fisheries, Japan [19]. The monitoring data of pests and diseases collected and preserved by the Ministry of Agriculture, Forestry and Fisheries were used in our study. The crop damages caused by major pests and diseases have been monitored as a national pest monitoring campaign for more than 75 years in Japan [19], wherein the abundances of pests and diseases are surveyed by all 47 prefectural monitoring stations monthly at one or a few monitoring spots to forecast the severity of damages in each prefecture. The abundance of a single pest or disease was measured by one or more suitable measurement units according to its characteristics, such as the individual number of a pest per leaf, the emerging area of a disease per plant, and the occurring area in the research field. We selected the exact numeric values of any measurement units from the datasets while discarding several ranked and presence/absence data. Consequently, each record contained crop name, and pest or disease names, monitoring unit, year, month, prefecture and the observed value. If the occurring area was considered as the measurement unit, the observed value was divided by the total planted area. We obtained total 43 monitoring units with 24 pests and diseases for cucumber, 75 of 29 for eggplant, 49 of 22 for tomato and 36 of 14 for strawberry (electronic supplementary material, table S1).

To consider the effect of meteorological and geographical factors, we incorporated temperature, precipitation and geographical information into the statistical models and ML methods as explanatory variables. We downloaded the records of the monthly average temperature and total precipitation for the duration of monitoring of all datasets from the website of the Japan meteorological agency (https://www.data.jma.go.jp/obd/stats/etrn/). We used the latitude and longitude of each prefectural office location as geographical information downloaded from the website of the geospatial information authority of Japan (https://www.gsi.go.jp).

We then combined the monitoring data and the meteorological and geographical information by year, month and prefecture. Each record consisted of crop, pest or disease names, monitoring unit, year, month, prefecture, monthly average temperature, monthly total precipitation, latitude, longitude and the monitored value of the pest or disease. We obtained total 85 222 records of 203 datasets that differed in measurement unit, pest and crop.

### 2.2. Model-fitting, validation and standardization processes

To compare the prediction performances of those methods using a variety of datasets, we set up a common calculation process for all methods. At first, we randomly divided each dataset into ten subsets. We used the subsets to perform a ten-fold cross-validation against all methods, wherein a model was fitted with the nine subsets and validated with the remaining subset. The monthly average temperature, monthly total precipitation and geographical information were used as explanatory variables. This process was performed ten times by switching the ten subsets in order. The value of RMSE between the observed and predicted values was calculated during each validation. An average of ten RMSE values was used as the model's prediction performance. Then, we also prepared null datasets by randomizing the sequence of years, months and prefectures, combined with meteorological and geographical information, of each original dataset. Similarly, we prepared ten subsets from a null dataset, assigned them to all methods, and calculated the RMSE values by using a ten-fold cross-validation. We repeated this procedure 100 times and got 100 RMSE values for each dataset.

We converted the RMSE values to *z* score as a standardized value, indicating the goodness of a model fit compared with null models. The *z* score is the standardized distance from the mean value of null models because it depends on the input values, as follows [20,21]:

$$z=\;\frac{{\mathrm{mean}}_{\mathrm{obs}}-{\mathrm{mean}}_{\mathrm{nulls}}}{\sigma_{\mathrm{nulls}}},$$

where mean_obs_ was the mean value of 100 RMSEs of ten-fold cross-validation, and mean_nulls_ and *σ*_nulls_ were the mean value and the standard deviation of RMSEs from null datasets, respectively. The value of the *z* score should be negative when the estimated model can produce better-predicted values than null models and should become smaller with the predicted values moving closer to the observed values and farther from null datasets.

### 2.3. Multiple linear regression model

A simple multiple regression model was assumed:

$$\mathrm{Incidence}=a_{\mathrm{Temp}}Temp+a_{\mathrm{prec}}Precip+a_{\mathrm{lati}}Lati+a_{\mathrm{longi}}Longi,$$

where *a*_temp_, *a*_prec_, *a*_longi_ and *a*_lati_ were coefficients of the monthly average temperature (*Temp*), monthly total precipitation (*Precip*), longitude (*Longi*) and latitude (*Lati*), respectively, and were common among prefectures. No interaction effects were considered.

### 2.4. Bayesian state-space model

We prepared a simplest possible Bayesian model to ensure that the random data sampling and parameter estimations using the Markov chain Monte Carlo method worked efficiently even with a small dataset. We assumed that the monthly dynamics of a pest or disease in a year differed among prefectures. The dynamics in a prefecture were minorly skewed from the central and general dynamics, which were the hypothetical dynamics averaged across the prefectures, in response to the monthly average temperature and total precipitation, latitude and longitude of the prefecture. We assumed a hypothetical zero month before January and defined its value as zero, denoting its estimated abundance. Model formulations are as follows.

μ[m,p]: the estimated abundance in a month, *m* (integer, 0 ≤ *m* ≤ 12), in a prefecture, *p* (natural number, 1 ≤ *p* ≤ 47)

α[m]: the estimated abundance in a month, *m*, of the central dynamics of a pest or disease

λ[m,p]: differential from the central abundance in a month, *m*, and a prefecture, *p*:

$$\lambda [m,p]=a_{\mathrm{temp}}Temp[m,p]+a_{\mathrm{prec}}Precip[m,p]+a_{\mathrm{longi}}Longi[p]+a_{\mathrm{lati}}Lati[p],$$

where *a*_temp_, *a*_prec_, *a*_longi_ and *a*_lati_ are coefficients. Each coefficient value was determined as a relative effect of each explanatory variable on the prefecture-specific effect, *λ*. The estimated abundance value in January in a prefecture, *p*, was *μ*[1, *p*], sampled from a normal distribution with the mean value. The central dynamics of January *α*[1] and a differential from the central dynamics in January in a prefecture *p*, *λ*[1, *p*], were added to the estimated abundance value of the zero month of a prefecture *p*, *μ*[0, *p*] (= 0), and the standard deviation *σ_μ_*, as follows:

$$\mu [1,p]∼Normal(\mu [0,p]+\alpha [1]+\lambda [1,p],\sigma_{\mu }).$$

The abundance value in the subsequent month, *m* + 1, in a prefecture *p*, *μ*[*m* + 1, *p*], was sequentially determined using the abundance value in the previous month, *μ*[*m*, *p*], the monthly change of the central dynamics, *α*[*m*], and the prefecture-specific effect, *λ*[*m*, *p*], as follows:

$$\mu [m+1,p]∼Normal(\mu [m,p]+\alpha [m]+\lambda [m,p],\sigma_{\mu }).$$

The observed abundance value, *Abundance*[*n*], of a record, *n*, was sampled from a normal probability distribution with the mean value, *μ*[*m*, *p*], and a standard deviation, *σ*_obs_, as follows:

$$Abundance[n]∼Normal(\mu [Month[n],Pref[n]],\sigma_{\mathrm{obs}}).$$

Using the above calculations, we estimated the values of 12 months in 47 prefectures even with several missing values in a dataset. This Bayesian state-space model was commonly used for all datasets of pests and diseases.

### 2.5. Machine learning

We used seven ML algorithms, namely, lasso, elastic net, DT, RF, kNN, SVM and NN, to predict the abundance of each pest and disease using the four explanatory variables, namely monthly average temperature, monthly total precipitation, longitude and latitude, at each location (i.e. prefecture). Prior to modelling, each of the four variables was rescaled with a mean value of zero and a standard deviation of one. Lasso, elastic net, DT, RF, kNN and SVM models were created using scikit-learn package version 0.32.2 [22], and NN models using PyTorch package version 1.6.0 [23]. The ten subsets described in the dataset preprocessing subsection were used for ten-fold cross-validation to evaluate model performance (i.e. RMSE). During the model training processes, training subsets were further split into two subsets to perform two-fold cross-validation to determine the hyper-parameters of the models using grid search. Detailed model structures (e.g. number and type of hyperparameters) of each ML algorithm and the ranges for grid search we set are shown in the electronic supplementary material.

### 2.6. Model comparison

To compare the *z* score among the nine methods, we used a linear mixed model in which the response variable was the *z* score, the explanatory variable was a method type, and the dataset was a random effect. Then to examine the relationship between data size and *z* score, we fitted linear and nonlinear models to the results. In a linear regression model, the explanatory variable was the number of records in a dataset, and the response variable was the *z* score. We then used two nonlinear models, where one was a quadratic equation with three parameters, *a*_1_, *a*_2_ and *a*_3_, as follows:

$$z=a_{1}x^{2}+a_{2}x+a_{3},$$

where *z* is the *z* score and *x* is the number of records. The second model was an asymptotic curve with three parameters, *b*_1_, *b*_2_ and *b*_3_, as follows:

$$z=\;b_{1}-\exp(\;b_{2}x+b_{3}),$$

where *b*_1_ was the asymptotic value, *b*_2_ determined the shape of the curve, and *b*_3_ moved the curve in the *x*-axis direction. We estimated the parameter values using the least square method. We compared the value of the AIC of the three regression models and selected the one with the minimum AIC value. We also checked the significance of the coefficients of explanatory variables to examine the effect of data size on the *z* score.

To compare the prediction tendency of the nine methods, we performed cluster analysis using a two-dimensional matrix that arranged *z* scores in rows of methods and columns of datasets. We separated the methods into groups based on the results.

To examine the effects of data size and bias on the *z* score, we calculated *F* values by dividing the between-prefecture variance of the pest abundance using the within-prefecture variance. When the abundance of a pest has been monitored in limited prefectures and limited months, the between-prefecture variance and the *F* value should be larger. To examine if the *z* score was affected by the *F* value, the method, and the number of records, we used a linear mixed model in which the method, *F* value, and the number of records were explanatory variables, the *z* score was the response variable, and the dataset was a random effect. Interactions of the explanatory variables were considered.

### 2.7. Analysis environment

Data splitting, RMSE standardization and model comparison using a linear mixed model and AIC values were performed on an open statistical software, R version 4.1.1 [24], on Microsoft Windows 10 Pro. Of the nine methods, Bayesian and multiple linear models were performed by R on Windows, while the other seven methods were done by Python 3.6.9 on Ubuntu 20.04. We used the package ‘rstan’ based on Stan software [25] on R to fit a Bayesian model to the dataset. In a model-fitting process using a Markov chain Monte Carlo (MCMC) sampler of Stan, the iteration number of sampling was 2000, and that of warmup was 500. This process was repeated four times. We then sampled 2000 matrices with 12 rows (months) and 47 columns (prefectures) from the estimated model with posterior parameter distributions. We used the ‘lme4’ package on R to fit the data to a linear mixed model [26].

## 3. Results

The maximum and minimum numbers of records in a single dataset were 2033 and 1, respectively. The distribution of the record number is shown in electronic supplementary material, figure S1. The median value of the record number in all 203 datasets was 128, 537 in cucumber, 114 in eggplant, 110 in strawberry and 135 in tomato. Three datasets had only a single record. The number of records, months, and prefectures with any records in each dataset are listed in electronic supplementary material, table S1.

Most methods, such as 10-fold cross-validation, failed to estimate the parameter values, and did not work when records were too few and most abundance records were zero. We excluded the results that were unable to estimate *z* scores (e.g. N.A.). The number of *z* scores finally included were 175 in Bayesian model, 187 in the linear model, 187 in lasso, 178 in elastic net, 187 in DT, 187 in RF, 172 in kNN, 186 in SVM and 187 in NN out of 203 datasets.

**Figure 1.**
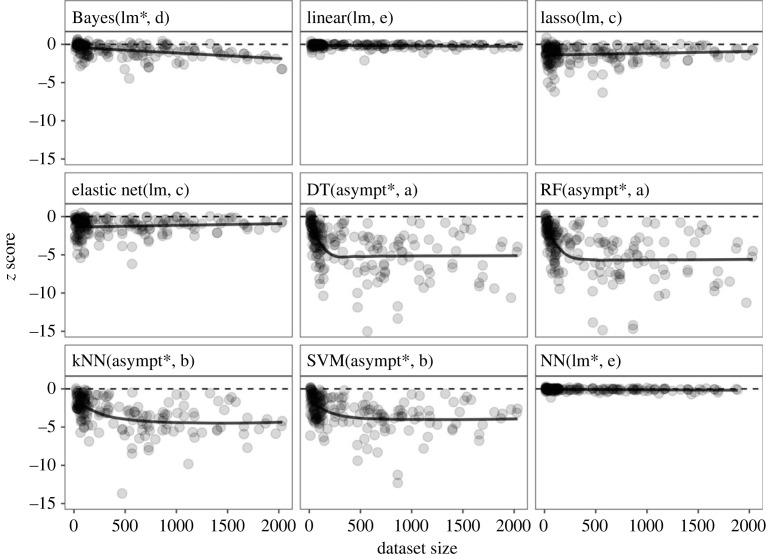
The value of standardized RMSE, *z* score, plotted against the number of records in a dataset when Bayesian model, linear model, lasso, elastic net, DT, RF, kNN, SVM or NN was used for each dataset (grey circle). A regression line (black line) was drawn when the AIC value of a linear model (lm) was the minimum, while a regression curve was drawn when that of an asymptotic curve (asympt) was the minimum. An asterisk (*) indicates the significance of the coefficient (*p* < 0.05).

To compare the prediction performance between the nine methods, we examined if *z* score values differed between them using a linear mixed model, with explanatory variable as method and random effect as dataset, and found a significant difference (*F* = 221.96, *p* < 0.001, figure 1; electronic supplementary material, figure S2). *Post hoc* multiple comparisons showed that the *z* scores of RF and DT were the smallest, followed by those of kNN and SVM at the second, lasso and elastic net at the third, and those of linear model and NN were the largest (figure 1; electronic supplementary material, figures S2 and S3).

We then examined if a relationship between *z* scores and the number of records was linear, quadratic or asymptotic, and found that the minimum AIC value was observed in either a linear regression or an asymptotic curve model in the nine methods (figure 1 and table 1; electronic supplementary material, figure S3). In Bayesian model, linear model, lasso, elastic net and NN, the AIC value of the linear model was smaller than that of the asymptotic model, with vice versa in DT, RF, kNN and SVM. The AIC value of a quadratic model was not the minimum in any method (electronic supplementary material, table S2). The significance of the coefficient value was observed in Bayesian model, DT, RF, kNN, SVM and NN. The *z* score linearly decreased with the number of records in Bayesian model and decreased but converged to the asymptotic value in DT, RF, kNN and SVM (figure 1; electronic supplementary material, figure S3).

**Table 1.** The AIC values when linear regression or asymptotic curve model was fitted to the number of records and the *z* scores calculated using one of the nine algorithms: Bayesian model, linear model, lasso, elastic net, DT, RF, kNN, SVM and NN. The *F* and *p* values indicate the result of the model comparison.

model	AIC		ANOVA		
	linear	asymptotic	*F* value	*p* value	
Bayes	418.6	NA	41.38	< 0.001	***
linear	50.1	51.9	3.06	0.0823	
lasso	591.0	592.2	2.11	0.1481	
elastic net	585.6	588.9	1.84	0.1762	
DT	899.2	871.1	32.17	< 0.001	***
RF	907.6	880.5	30.92	< 0.001	***
kNN	720.3	703.1	19.90	< 0.001	***
SVM	779.4	760.3	21.97	< 0.001	***
NN	−125.4	−123.4	7.08	0.0085	**

**Figure 2.**
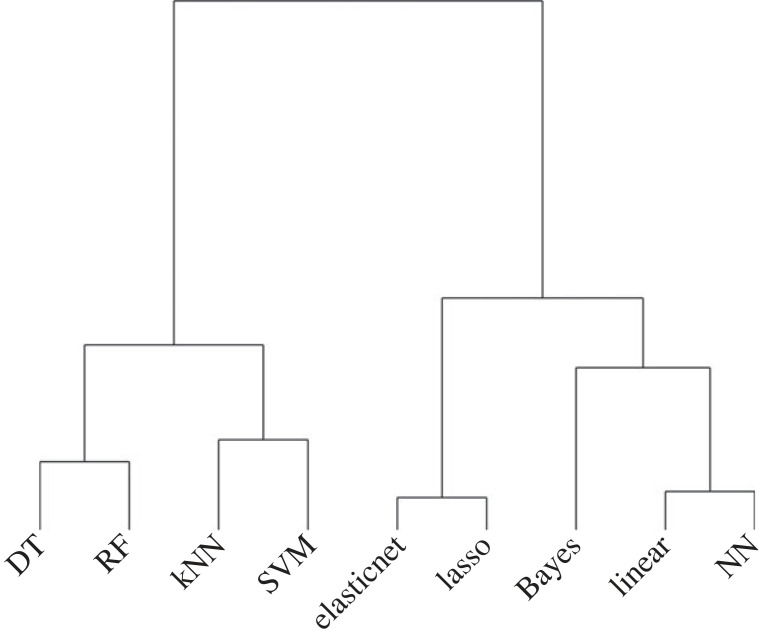
A dendrogram of the nine methods produced by cluster analysis. The methods used are Bayesian model, linear model, lasso, elastic net, DT, RF, kNN, SVM or NN.

The cluster analysis dendrogram showed that the nine methods were divided into two groups (figure 2), including DT, RF, kNN and SVM in one and lasso, elastic net, Bayesian model, linear model and NN in the other. The first classification group was further divided into two sub-groups (clusters A and B), wherein cluster A consists of DT and RF and contains methods based on decision tree, while cluster B contains kNN and SVM. The regression group was also divided into two sub-groups (clusters C and D), wherein cluster C contains lasso and elastic net and selects explanatory variables based on a regularization term (*L*_p_ norm), while cluster D contains Bayesian model, linear model and NN, and uses all explanatory variables. These results are consistent with those of principal component analysis (electronic supplementary material, figure S4).

**Figure 3.**
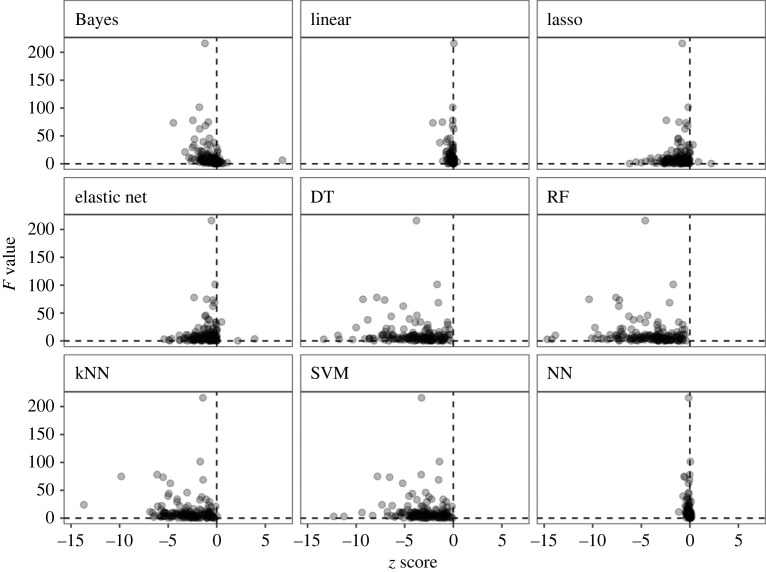
*F* value, indicating the data bias of between-prefecture to within-prefecture, plotted against the standardized RMSE, *z* score, when Bayesian model, linear model, lasso, elastic net, DT, RF, kNN, SVM or NN was used.

We examined the effects of method, record number and data bias on *z* score using a linear mixed model, and found that pest abundance records in a dataset were often biased to limited prefectures (electronic supplementary material, table S1). To evaluate the data bias, we calculated *F* values by dividing the between-prefecture variance of the pest abundance by the within-prefecture variance. The effects of method were found to be significant (*F* = 64.31, *p* < 0.001) and the record number (*F* = 17.40, *p* < 0.001) on *z* score, but not that of *F* value (*F* = 2.31, *p* = 0.13). The interaction effects of method and *F* value (*F* = 3.07, *p* = 0.0020), and method and the record number (*F* = 19.09, *p* < 0.001) were significant, but that of *F* value and the record number was not (*F* = 3.74, *p* = 0.055). The interaction effect of the three characteristics was significant (*F* = 4.67, *p* < 0.001). The *z* score differed between methods and then decreased with increase in the record number. The effect of the record number on *z* score was different among the methods. These findings are consistent with the results mentioned above. Further, this analysis showed that the effect of *F* value, as an index of data bias, on *z* score differed among different methods (figure 3).

## 4. Discussion

The four best methods, DF, DT, kNN and SVM, are all efficient at classification despite their different algorithms [27]. Our results suggest that classification methods of ML can be a powerful and flexible approach to predict pest occurrence and abundance, depending on the dataset characteristics. In other words, these simple ML methods can perform better than statistical models with some structures based on prior information, such as the Bayesian model. Among the four clusters, cluster A, which includes DT and RF, exhibited the best performance. These two algorithms are similar. The random forest comprises small decision trees. The best model built by either of these methods processes the values of explanatory variables according to a unidirectional flow chart resembling a tree. Cluster B, which includes kNN and SVM, exhibited the second-best performance. SVM makes the prediction value using an insensitive loss function, while kNN predicts the attribute value using given feature values and existing neighbouring feature values. These two methods differ in algorithm but commonly explore a flexible decision boundary that best explains the data.

The classification methods' superiority suggests a categorical, rather than linear, relationship between explanatory and response variables. For example, the abundance records of a pest or disease were geographically biased to limited prefectures in a dataset. Similar to classification methods, predicting by prefecture would be more accurate than by environmental gradients. We found that the effect of between-prefecture bias on *z* score differed between the nine methods. In figure 3, several points were found to be scattered in the area with smaller *z* scores and larger *F* values in the case of RF, DT, SVM and kNN. These results suggest that the classification methods showed better prediction performance when the records were biased between prefectures, and the existence of a data bias.

There can be several reasons for the biased datasets. One reason is the biased and spotted distribution and occurrence of a pest or disease. Some pests’ and diseases’ occurrence is not dependent on macro-scale environmental and geographical gradients [28] but on micro-scale ones, such as soil moisture and surrounding landscape. Certain thrips, whiteflies, aphids and mites often occur in spotted areas and tend to be removed as soon as possible. In these cases, the recorded distributions of the pests should have been spotted. Another reason is that the monitoring is done in limited prefectures and periods (electronic supplementary material, table S1). Only 54 of 203 datasets (26.6%) have records of more than 20 out of 47 prefectures. Limited human resource at the prefectural monitoring stations prevents completion of surveys at all monitoring units in the four crops. Prefectural surveyors must carefully select the limited measurement units of the target pests and diseases at higher risk of outbreak in their prefectures and exclude those with lower risks. Similarly, they must select the limited seasons of higher likeliness of occurrence. These spatio-temporally limited records can also conceal the environmental and geographical gradients and correlations. Several similar datasets are missing parts of pest monitoring records and have not been used. This study shows that the classification methods can be applied to such sparse and biased datasets.

Meanwhile, linear regression methods (linear model, Bayesian model, lasso, and elastic net) were inferior to classification methods. Results are consistent with those of a previous study [12]; RF and kNN were better predictors than multiple linear model. Lasso and elastic net were separated from linear model by cluster analysis, which may be caused by the absence of the penalty term. Bayesian and linear models use all explanatory variables when determining the best performing model, while lasso and elastic net remove ineffective explanatory variables according to the penalty term. Lasso and elastic net can remove more explanatory variables with a smaller dataset. This interpretation can also be applied to NN being grouped with Bayesian and linear models in cluster analysis. We defined only two layers in NN to ensure that it can build a model with a small dataset (see electronic supplementary material). This definition makes NN similar to a simple multiple regression model.

The decreasing pattern of *z* score with increasing dataset size differed between the classification methods and Bayesian model. The Bayesian model was the only method of which the prediction performance continued to improve as the data size increased because its best fit was a linear model with a significantly negative coefficient value. It suggests that environmental gradients, including temperature and precipitation, become clearer with the increase in data size, facilitating their detection by Bayesian model. On the contrary, results of DT, kNN, RF and SVM showed that the prediction performance improved but asymptotically converged to a certain value with the increase in data size. As mentioned above, this pattern should be due to the nonlinear relationship between the explanatory and response variables. A linear model or others may be the best fit in the classification methods if the dataset size is far larger than those used in this study. The effect of data size on *z* scores of the other regression models, linear model, lasso and elastic net, was insignificant. These models did not consider any seasonal occurrence patterns of the pest abundance, while Bayesian model did in a state-space model. These results suggest that seasonality should be considered in a model when using regression models for analysing extensive time-series data. While this study demonstrated that some classification methods outperformed other statistical models when provided with biased datasets, it does not guarantee their suitability for real-world alerting systems. Bayesian and other statistical models would still be valuable for the localized subset of the dataset in cases where data bias is detected.

## 5. Conclusion

We compared statistical and ML methods that have been used to predict the abundance of a crop pest or disease using a large number of datasets. We found that the classification methods of ML were superior to linear regression ones due to the missing records and their bias in a dataset. We suggest that the classification methods can be a favourable approach to making a prediction, even with scarce and biased datasets. Bayesian model was the only method for which the prediction performance was found to increase linearly with increasing dataset size, suggesting that the biologically interpretable structure in Bayesian model worked well to detect the environmental and geographical gradients. Our results suggest that even though ML methods are gaining popularity, they are unsuitable for all datasets and purposes. Selecting appropriate methods depending on the dataset characteristics and scientific rationale is essential to predict or understand the mechanisms.

## Data Availability

Data and relevant R and Python codes for this research work are stored in GitHub (https://github.com/ShigekiKishi/Statisticalmodels_vs_Machinelearning) and have been archived within the Zenodo repository (https://doi.org/10.5281/zenodo.8010516) [29] and the Open Science Framework repository (https://osf.io/g72ac/). All datasets we used in this study are available at the Open Science Framework repository (https://osf.io/g72ac/). We have summarized the datasets in electronic supplementary material, table S1, wherein the crop name, pest or disease name, survey unit, the number of records, the number of unique months, and the number of unique prefectures are described for each. R and Python codes for reproducing the results in this paper are available at the Github repository (https://github.com/ShigekiKishi/Statisticalmodels_vs_Machinelearning). The data are provided in electronic supplementary material [30].
